# Multi-GNSS Precise Point Positioning with UWB Tightly Coupled Integration

**DOI:** 10.3390/s22062232

**Published:** 2022-03-14

**Authors:** Zhenchuan Huang, Shuanggen Jin, Ke Su, Xu Tang

**Affiliations:** 1 School of Communication and Information Engineering, Shanghai University, Shanghai 200444, China; huangzhenchuan@whu.edu.cn; 2 Shanghai Astronomical Observatory, Chinese Academy of Sciences, Shanghai 200030, China; suke17@mails.ucas.ac.cn; 3 School of Surveying and Land Information Engineering, Henan Polytechnic University, Jiaozuo 454000, China; 4 School of Remote Sensing and Geomatics Engineering, Nanjing University of Information Science and Technology, Nanjing 210044, China; xu.tang@nuist.edu.cn

**Keywords:** precise point positioning (PPP), ultra-wideband (UWB), time difference of arrival (TDOA), tightly coupled integration

## Abstract

Global Navigation Satellite Systems (GNSSs) can provide high-precision positioning services, which can be applied to fields including navigation and positioning, autonomous driving, unmanned aerial vehicles and so on. However, GNSS signals are easily disrupted in complex environments, which results in a positioning performance with a significantly inferior accuracy and lengthier convergence time, particularly for the single GNSS system. In this paper, multi-GNSS precise point positioning (PPP) with tightly integrating ultra-wide band (UWB) technology is presented to implement fast and precise navigation and positioning. The validity of the algorithm is evaluated by a set of GNSS and UWB data. The statistics indicate that multi-GNSS/UWB integration can significantly improve positioning performance in terms of the positioning accuracy and convergence time. The improvement of the positioning performance for the GNSS/UWB tightly coupled integration mainly concerns the north and east directions, and to a lesser extent, the vertical direction. Furthermore, the convergence performance of GNSS/UWB solution is analyzed by simulating GNSS signal interruption. The reliability and robustness of GNSS/UWB solution during GNSS signal interruption is verified. The results show that multi-GNSS/UWB solution can significantly improve the accuracy and convergence speed of PPP.

## 1. Introduction

Global Navigation Satellite Systems (GNSSs), including USA’s Global Positioning System (GPS), Russia’s GLObal NAvigation Satellite System (GLONASS), China’s BeiDou Navigation Satellite System (BDS) and Europe’s Galileo [1], have been widely used in various fields, such as navigation and timing, geodesy, seismic monitoring and gravity field [2,3,4,5,6]. However, GNSS positioning performance becomes seriously degraded in a challenging GNSS environment with few observing satellites and multipath effects. To further improve the reliability and availability of GNSS positioning, multi-sensors have been integrated into the GNSS [7]. Ultra-wideband (UWB) has a high transmission rate, strong anti-interference ability and multipath capability [8]. It applies a unique pulse signal for the short-distance communication, with a frequency range of 3.1~10.6 GHz and a pulse width of nanosecond or sub-nanosecond level [9]. Some research on the integration of GPS and UWB has been carried out, which could lead to the achievement of excellent positioning accuracy in GPS-challenging environments. The loosely coupled integration was employed in early GPS/UWB integration research [10,11,12]. The approach is computationally low when GPS is working, but it fails when GPS measurements are not available. Following this, some scholars proposed the GPS/UWB tightly coupled integration, which can increase the redundancy of observations and improve the reliability of the positioning system. For instance, Chui et al. [13] firstly applied GPS/UWB tightly coupled integration in a complex urban environment, which indicated that the positioning error in the east direction was reduced from 5.43 cm to 2.61 cm. Chui et al. [14] also investigated the tightly coupled integration modeling of differential GPS (DGPS) and UWB, although it can only achieve meter-level accuracy in a kinematic environment. Moreover, tightly coupled integration modeling of GPS and UWB for precision applications was provided in MacGougan et al. [15]. The results showed that the ambiguity float solution had increased from half a meter accuracy to sub-meter accuracy, and the convergence time of the ambiguity fixed solution reduced. Thereafter, MacGougan et al. [16] applied GPS/UWB tightly coupled integration in a difficult realistic environment, and maintained sub-meter accuracy. Recently, Shen et al. [17] performed the DGPS/UWB tight integration in RTK for the emerging intelligent transportation systems, but the root mean square (RMS) error of the proposed method was still large.

In recent years, the GPS/UWB integration methods based on GPS Single Point Positioning (SPP) and Real-time Kinematic (RTK) have been applied successively due to the simple implementation of SPP and the high accuracy of RTK [16,18,19,20]. However, the SPP algorithm can only provide meter-level accuracy and cannot meet high accuracy requirements. Although the RTK method can provide centimeter-level accuracy and rapid convergence, it requires dedicated base stations or a dense network coverage [21]. The positioning performance is highly influenced by the distance between users and base station [22]. Precise Point Positioning (PPP) technology is introduced to overcome these disadvantages [23], which is a flexible and high-precision single-point positioning mode using the GNSS pseudo-range and carrier phase observations as well as satellite precise orbit and clock products provided by International GNSS Service (IGS) [24]. It relies only on one receiver for a user to realize the absolute positioning. The main factors affecting the accuracy of PPP are the number of satellites, spatial geometry structure between user and satellites, and the quality of observations [25]. For GPS positioning, at least four satellites are necessary in open-sky conditions. Once the GPS signal is blocked or subject to multipath interference, the number of available satellites is likely to be less than four and the corresponding availability and accuracy degrades dramatically. Hence, the integration of the multi-constellation GNSS is an effective and direct way by which to improve PPP’s performance [26]. The existing literature has demonstrated that integrating both GPS and GLONASS or other satellite navigation systems can increase the number of observation satellites and significantly improve the positioning performance [27,28,29]. Nevertheless, PPP takes a long time to converge before reaching centimeter-level accuracy [30].

The limitation of GNSS positioning applications is still challenging, such as the long convergence time, the poor satellite availability and continuity in complex scenes including city urban, indoor-outdoor scenes, tunnel and so on. To solve the problem of seamless indoor and outdoor positioning, GNSS PPP has been tightly integrated with UWB in complex environments to enhance the GNSS positioning performance. UWB technology has a high sampling frequency and can achieve centimeter-level positioning accuracy over a short time, which can provide initial constraints for GNSS and accelerate PPP convergence. The integration of UWB and GNSS can enhance model strength, effectively compensate for the shortcomings of GNSS system, and improve system performance in terms of accuracy, continuity and availability.

In this paper, a tightly coupled integration method of multi-GNSS and UWB based on PPP model is proposed, and the corresponding integration model is presented and implemented. Simultaneously, the positioning performance of the GNSS/UWB solutions is evaluated by comparing to the GNSS-only solutions. The mathematical models of the single or combined navigation system are described in detail in Section 2. Then, a set of the real-measured GPS, GLONASS, and UWB data is processed in GNSS-only and GNSS/UWB modes, respectively. The positioning accuracy and convergence performance of the multi-GNSS/UWB tightly coupled integration under different scenarios are assessed and analyzed in Section 3. Finally, conclusions are provided in Section 4.

## 2. Methods and Models

In this section, GNSS and UWB positioning systems are introduced in subsections at first. Then, the multi-GNSS/UWB tightly coupled integration based on PPP algorithm is described in detail.

### 2.1. GNSS Positioning System

The basic principle of GNSS positioning is to determine the position of the receiver by measuring the distance of the GNSS satellite *s* and the GNSS receiver *r*. Assuming that the satellite clock $t^{s}$ and receiver clock $t_{r}$ are synchronized, there is still a clock difference Δt between them, so the measured distance $p_{r}^{s}$ is called the pseudo-range. The pseudo-range measurement equation is given as follows:

(1) $$p_{r}^{s}=\rho_{r}^{s}+c\cdot \Delta t$$

where $\rho_{r}^{s}=\sqrt{{\left(X-X_{i}^{s}\right)}^{2}+{\left(Y-Y_{i}^{s}\right)}^{2}+{\left(Z-Z_{i}^{s}\right)}^{2}}$, is the geometry distance between the receiver and the satellite i; $\left(X_{i},Y_{i},Z_{i}\right)$ is the three-dimensional coordinates of the satellite i in the Earth center Earth fixed (ECEF) coordinate system; (X,Y,Z) is the ECEF coordinates of the receiver; *c* = 299,792,458 m/s, is the speed of light.

The general GNSS PPP observation equations should consider additional error sources. The corresponding observation equations for the raw pseudo-range and carrier phase on frequency *j* are written as follows [31]:

(2) $$\left\{ \begin{array}{l} p_{r,j}^{s}=\rho_{r}^{s}+c\left(t_{r}-t^{s}\right)+c\left(d_{r,j}-d_{j}^{s}\right)+T_{r}^{s}+I_{r,j}^{s}+\varepsilon_{p_{r,j}^{s}},\varepsilon_{p_{r,j}^{s}}\sim \left(0,\sigma_{p_{r,j}^{s}}^{2}\right)\\ \varphi_{r,j}^{s}=\rho_{r}^{s}+c\left(t_{r}-t^{s}\right)+\lambda_{j}\left(b_{r,j}-b_{j}^{s}\right)+T_{r}^{s}-I_{r,j}^{s}+\lambda_{j}N_{r,j}^{s}+\varepsilon_{\varphi_{r,j}^{s}},\varepsilon_{\varphi_{r,j}^{s}}\sim \left(0,\sigma_{\varphi_{r,j}^{s}}^{2}\right) \end{array}\right.$$

where $T_{r}^{s}$ is the tropospheric delay; $d_{r,j}$ and $d_{j}^{s}$ are the uncalibrated code delays (UCDs) with respect to the receiver and satellite; $b_{r,j}$ and $b_{j}^{s}$ are the uncalibrated phase delays (UPDs) with respect to the receiver and satellite; $I_{r,j}^{s}$ is the ionospheric delay; $\lambda_{j}$ is wavelength of the carrier phase on frequency *j*; $N_{r,j}^{s}$ is the float ambiguities; $\varepsilon_{p_{r,j}^{s}}$ and $\varepsilon_{\varphi_{r,j}^{s}}$ are the pseudo-range and carrier phase observation noises including multipath.

The ionosphere-free (IF) combination can remove the is first-order ionospheric delay, which is widely applied in dual-frequency PPP model, and the observation equations of the IF combination can be expressed as [24]:

(3) $$\left\{ \begin{array}{l} P_{r,IF}^{s}=\frac{f_{1}^{2}}{f_{1}^{2}-f_{2}^{2}}P_{1}-\frac{f_{2}^{2}}{f_{1}^{2}-f_{2}^{2}}P_{2}\\ \Phi_{r,IF}^{s}=\frac{f_{1}^{2}}{f_{1}^{2}-f_{2}^{2}}\Phi_{1}-\frac{f_{2}^{2}}{f_{1}^{2}-f_{2}^{2}}\Phi_{2} \end{array}\right.$$

where $P_{r,IF}^{s}$ and $\Phi_{r,IF}^{s}$ are the IF combination of pseudo-range and carrier phase; $f_{1}$ and $f_{2}$ are the corresponding frequencies; $P_{1}$ and $P_{2}$ are the raw pseudo-range on frequency $f_{1}$ and $f_{2}$; $\Phi_{1}$ and $\Phi_{2}$ are the carrier phase on frequency $f_{1}$ and $f_{2}$.

With the precise clock products estimated with the corresponding IF combination and other corrected error sources available, the UCD can be corrected and the UPD can be absorbed by the offsets and ambiguities of the receiver clock [32]. Thereafter, we should consider the inter-system biases (ISB) arising from the hardware delays of different GNSSs. The corresponding IF combination of pseudo-range and carrier phase can be further simplified as:

(4) $$\left\{ \begin{array}{l} P_{r,IF}^{s}=\rho_{r}^{s}+c\cdot t_{r,G}+ISB_{G}^{sys}+M\cdot T_{r}+\varepsilon_{P_{r,IF}^{s}}\\ \Phi_{r,IF}^{s}=\rho_{r}^{s}+c\cdot t_{r,G}+ISB_{G}^{sys}+M\cdot T_{r}+\lambda_{r,IF}\cdot N_{r,IF}^{s}+\varepsilon_{\Phi_{r,IF}^{s}} \end{array}\right.$$

where *G* represents the GPS satellite, *sys* represents the BDS, GLONASS, or Galileo satellite. $ISB_{G}^{sys}=t_{r,sys}-t_{r,G}$, is the ISB of each system with respect to the GPS; $N_{r,IF}^{s}$ is the ambiguity parameter of the IF combination; M is the mapping function of the wet tropospheric delays; $\lambda_{r,IF}$ is the wavelength of the carrier phase of the IF combination; $\varepsilon_{P_{r,IF}^{s}}$ and $\varepsilon_{\Phi_{r,IF}^{s}}$ denote the observation noises of the IF combination.

For GPS and GLONASS data, the linearized observation equations of the IF combination can be written as follows:

(5) $$\{\begin{array}{l} p_{IF,G}=-u\cdot \delta p+c\cdot \delta t_{r,G}+M\cdot \delta T_{r}+\varepsilon_{p_{G}}\\ \varphi_{IF,G}=-u\cdot \delta p+c\cdot \delta t_{r,G}+\lambda_{IF}\cdot \delta N_{IF,G}+M\cdot \delta T_{r}+\varepsilon_{\varphi_{G}}\\ p_{IF,R}=-u\cdot \delta p+c\cdot \delta t_{r,G}+ISB_{G}^{R}+M\cdot \delta T_{r}+\varepsilon_{p_{R}}\\ \varphi_{IF,R}=-u\cdot \delta p+c\cdot \delta t_{r,G}+ISB_{G}^{R}+\lambda_{IF}\cdot \delta N_{IF,R}+M\cdot \delta T_{r}+\varepsilon_{\varphi_{R}} \end{array}$$

where $p_{IF}$ and $\varphi_{IF}$ denote the measurement residuals of the IF combination; u is the unit vector from the receiver to the satellite; $\delta p=\left[\begin{array}{ccc} dx & dy & dz \end{array}\right]$ is the positioning increment vector.

### 2.2. UWB Positioning Method

The Federal Communication Commission (FCC) defines UWB signal. Due to its high bandwidth, UWB can use short-pulse waveforms, which is beneficial when reducing multipath interference [33]. The UWB positioning methods include Two Way Time of Flight (TW-TOF), Time of Arrival (TOA), Received Signal Strength Indication (RSSI), Time Difference of Arrival (TDOA) and Angle of Arrival (AOA) [34]. This paper utilizes the TDOA-based method for the UWB positioning.

The TDOA approach estimates the position of the tag by measuring the arrival time difference between the tag signal to each of the known base stations. The time difference is multiplied by the speed of light to obtain the distance difference between the tag and base stations, thereafter the hyperbola equations are constructed. The intersection of the hyperbolas is the position of the tag. The TDOA algorithm does not need to synchronize the time of the tag and each base station, but needs to maintain time synchronization between the different base stations. The time of arrival equation can be written as:

(6) $$\sqrt{{\left(X_{i}-x\right)}^{2}+{\left(Y_{i}-y\right)}^{2}+{\left(Z_{i}-z\right)}^{2}}=c\cdot t_{i}$$

where $\left(X_{i},Y_{i},Z_{i}\right)$ is the three-dimensional coordinates of the base station i in the ECEF coordinate system; (x,y,z) is the three-dimensional coordinates of the estimated positioning tag; $t_{i}$ is the arrival time between the base station i and the positioning tag.

Using base station *1* with known coordinate as the reference base station, the TDOA observation equations read:

(7) $$c\cdot (t_{i}-t_{1})=\sqrt{{\left(X_{i}-x\right)}^{2}+{\left(Y_{i}-y\right)}^{2}+{\left(Z_{i}-z\right)}^{2}}-\sqrt{{\left(X_{1}-x\right)}^{2}+{\left(Y_{1}-y\right)}^{2}+{\left(Z_{1}-z\right)}^{2}}+\varepsilon_{i,1},\varepsilon_{i,1}\sim \left(0,\sigma_{uwb}^{2}\right)$$

where $\sigma_{uwb}^{2}$ is the a priori variance of the UWB measurements, which are mainly determined by the sensor performance and can be obtained by calibration or the prior statistical data.

The corresponding linearized TDOA observation equation can be expressed as:

(8) $$d^{uwb}=-v\cdot \delta p+\varepsilon_{d}$$

where $d^{uwb}$ is the TDOA measurements residuals; v is the difference between the unit vector of the tag to other base stations and the direction from the label to the reference base station.

### 2.3. GNSS/UWB Integration Method

#### 2.3.1. GNSS/UWB Integration Model Based on EKF

The Kalman filter method is used for the GNSS/UWB integrated system in the paper. Owing to the observation equations being nonlinear, an extended Kalman filter (EKF) is adopted [35]. When both GNSS data and UWB data are available at epoch k, the GNSS/UWB tightly coupled integration model will be implemented.

The related observation model for Kalman filter can be expressed by following equation:

(9) $$z_{k}=H_{k}x_{k}+\varepsilon_{k},\varepsilon_{k}∼N\left(0,R_{k}\right)$$

where $H_{k}$ is the design matrix, which can be obtained from Equations (5) and (8); $R_{k}$ is the prior covariance matrix of the observation noise $\varepsilon_{k}$.

According to Equations (5) and (8), the measurement vector of the GNSS/UWB tightly coupled integration is written as:

(10) $$z_{k}={\left[\begin{array}{cccccccc} p_{m}^{G} & \varphi_{m}^{G} & \cdots & p_{n}^{R} & \varphi_{n}^{R} & \cdots & d_{i}^{uwb} & \cdots \end{array}\right]}^{T}$$

where *m*, *n* and *i* represent the *m*th GPS satellite, *n*th GLONASS satellite and *i*th UWB base station, respectively.

The state parameter vector for the GNSS/UWB tightly coupled integration can be expressed as:

(11) $$x={\left[\begin{array}{cccc} \delta p & \delta t_{r} & \delta T_{r} & \begin{array}{cc} \delta N_{IF,G} & \delta N_{IF,R} \end{array} \end{array}\right]}^{T}$$

where $\delta t_{r}=\left[\begin{array}{cc} \delta t_{r,G} & ISB_{G}^{R} \end{array}\right]$ is the receiver clock offset vector including the receiver clock and ISB for each individual system.

The optimal estimation of the state parameter vector can be obtained using one-step prediction and measurement update of Kalman filter. The prediction means time update, which is based on the following state transition model:

(12) $$\{\begin{array}{l} {\hat{x}}_{k,k-1}=\Gamma_{k,k-1}{\hat{x}}_{k-1}+w_{k-1}\\ P_{k,k-1}=\Gamma_{k,k-1}P_{k-1}\Gamma_{k,k-1}^{T}+Q_{k-1} \end{array}$$

where $\Gamma_{k,k-1}$ is the state transition matrix; ${\hat{x}}_{k,k-1}$ and $P_{k,k-1}$ denote the predicted state parameter vector and covariance matrix; $Q_{k-1}$ is the covariance matrix of the process noise.

The measurement update is used to correct the state vector and performed only at the epochs where GNSS measurements and UWB measurements are available. The state parameters and the corresponding covariance matrix are updated:

(13) $$\left\{ \begin{array}{l} K_{k}=P_{k,k-1}H_{k}^{T}{\left(H_{k}P_{k,k-1}H_{k}^{T}+R_{k}\right)}^{-1}\\ {\hat{x}}_{k}={\hat{x}}_{k,k-1}+K_{k}\left(z_{k}-H_{k}{\hat{x}}_{k,k-1}\right)\\ P_{k}=\left(I-K_{k}\right)P_{k,k-1}{\left(I-K_{k}\right)}^{T}+K_{k}R_{k}{K_{k}}^{T} \end{array}\right.$$

where $K_{k}$ is the gain matrix at epoch time *k*, I is the identity matrix. In this paper, for mildly dynamic scenes, the position states are modeled as random walk processes with process noise in the integration model [16].

#### 2.3.2. Implementation of GNSS/UWB Algorithm

**Figure 1:**
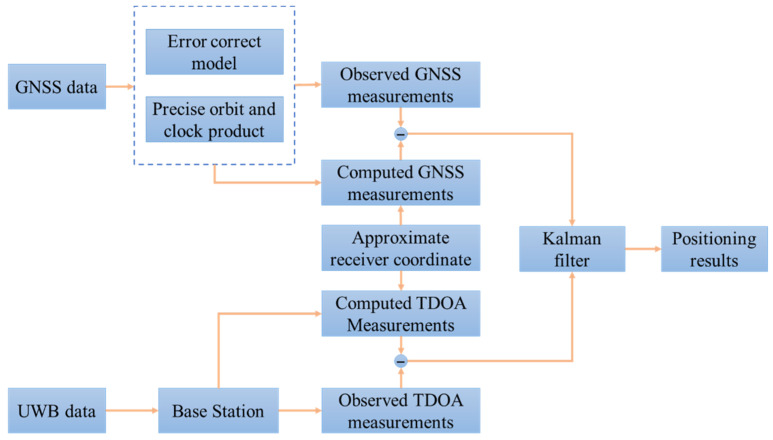
Framework of Global Navigation Satellite System (GNSS)/Ultra-wideband (UWB) tightly coupled integration algorithm.

As can be seen, Figure 1 shows the architecture of the multi-GNSS/UWB tightly coupled integration positioning system. GNSS data include the raw carrier phase and pseudo-range observations, and UWB data include the positions of the base stations and TDOA observations. The prior approximate receiver coordinate is obtained by an observation file. With the ephemeris of the GNSS satellites, the approximate position is applied in the calculation for the computed pseudo-range and carrier phase IF measurements. The pseudo-range and carrier phase IF measurements corrected by the error correction models and precise orbit and clock products are input with the computed GNSS measurements in the Kalman filter. Simultaneously, the TDOA measurements are computed by combining the known positions of base stations and the approximate receiver position. The observed UWB measurements are likewise input into the Kalman filter along with the computed TDOA measurements. Finally, the positioning result is achieved.

In GNSS data processing, the IF observations from GPS L1/L2, GLONASS G1/G2 signals are adopted and they have a sampling interval of 1 s. Phase center offset (PCO) and phase center variation (PCV) of GPS and GLONASS satellites are corrected using antenna files provided by IGS Center. Precise orbit and clock products provided by GeoForschungsZentrum (GFZ) are applied, which have a sampling interval of 5 min and 30 s, respectively. The modified Hopfield model is used to correct the tropospheric delay. Other error corrections are corrected by the corresponding models. In the UWB data processing, the sampling rate of UWB data is 10 Hz. The 3-sigma principle is applied to eliminate outliers of UWB data, and the non-line of sight (NLOS) errors are weakened in UWB data preprocessing [36]. For GNSS/UWB tightly coupled integration data processing, the approximate coordinate from the observation file is used as a prior for the EKF. Forward Kalman filter is applied to estimate the parameters. According to their prior statistical data, the initial standard deviation of the pseudo-range and carrier phase are 0.3 m and 0.003 m. The standard deviation of UWB measurements is set to 0.1 m, which is determined by the actual accuracy of the UWB range.

## 3. Experiments and Results

In this section, a set of GNSS and UWB data collected by a trolley test are processed to assess the positioning performance of the GNSS/UWB tightly coupled integration, in terms of the positioning accuracy and convergence time. In addition, convergence performance and positioning performance during GNSS signal interruptions are demonstrated by processing data in a dynamic mode.

### 3.1. Experimental Description

**Figure 2:**
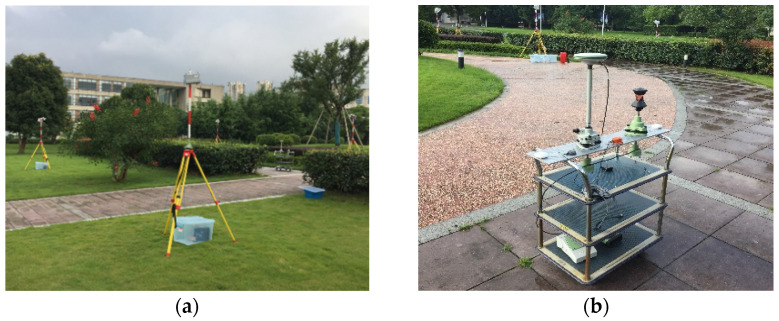
GNSS/UWB experimental scenarios and equipment (**a**) experimental environment; (**b**) Testing platform.

To verify the positioning performance of the GNSS/UWB tightly coupled integration model, a cart experiment was carried out at the University of Nottingham Ningbo, China. The experiment simulates the movement of a cart from indoors to outdoors. The cart was equipped with a UWB tag, a prism and a multi-GNSS receiver (LEICA GS10). The experimental environment and platform are shown in Figure 2. The approximate lever arm between the GNSS antenna and the UWB antenna was measured in advance and compensated for in the data-preprocessing process. Another multi-GNSS receiver (LEICA GR25) used for the GNSS base station was located less than 1 km away, and the position of the GNSS base station was known. The UWB base stations were set up on pre-surveyed locations with the same height. The maximum working distance of UWB base stations is about 20 m. The UWB equipment was provided by Sichuan Kunchen Technology Company.

**Figure 3:**
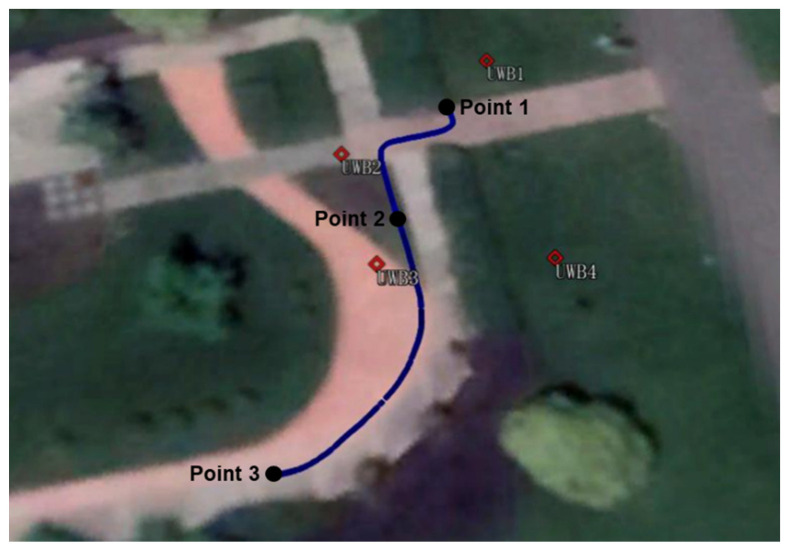
Platform the trajectory of the trolley experiment on Google Earth.

During the test, the trolley moved slowly along the southwest to the northeast direction with a maximum speed of 0.4 m/s. The experiment began at 6:00 (Universal Time Coordinated, UTC), and the time span of this test was approximately 2.5 h. The corresponding moving trajectory measured by a total station is shown in Figure 3. The whole trajectory is divided into the following stages:

1. To achieve convergence to the decimeter or centimeter level before the dynamic scenario, the trolley is stationary at Point 1 for about 1.5 h, when only GNSS signals are available;
2. The trolley moves from Point 1 to Point 2, simulating an indoor scene where the UWB signals are available and GNSS signals are unavailable;
3. The trolley is still static at Point 2 for about one hour, when the signals are both available, and the GNSS signals are interrupted once;
4. Finally, the trolley moves from Point 2 to Point 3, when the UWB signals are unavailable and the GNSS signals are available.

The GNSS/UWB tightly coupled integration model was realized by positioning-software developed by the authors. The RTK positioning solution calculated by RTKLIB software is used as the reference values in this study.

### 3.2. Positioning Accuracy

The static data was processed in static mode by artificially setting the cutoff elevation angle to simulate GNSS-only or GNSS/UWB operation in a complex urban environment.

#### 3.2.1. Static Results with an Open-Sky Condition

**Figure 4:**
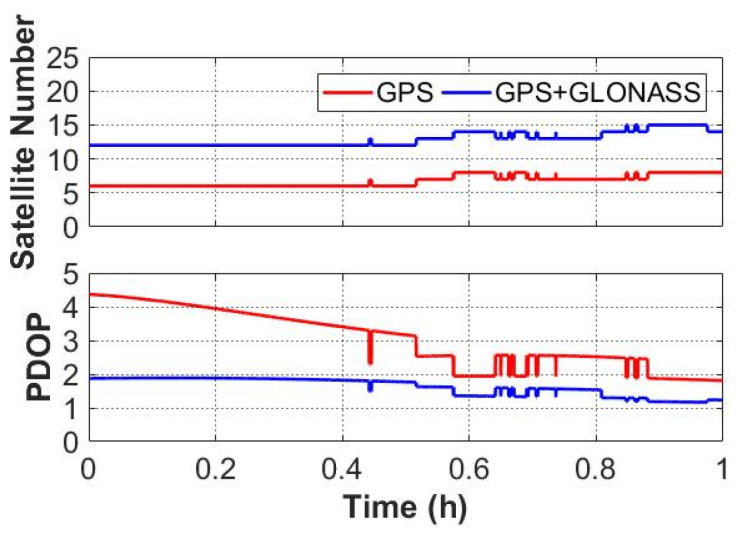
The number of the observed Global Positioning System (GPS) and GLObal NAvigation Satellite System (GLONASS) satellites (**top**) and PDOP values (**bottom**).

**Figure 5:**
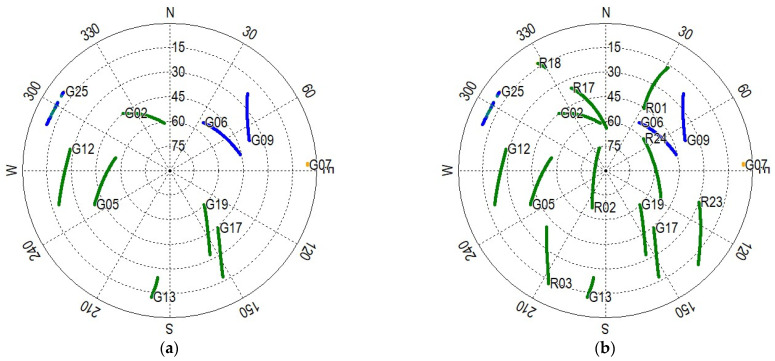
Sky plots of the GNSS satellites (the yellow indicates that only L1 is available, the green indicates that L1 and L2 are available, and the blue indicates that L1, L2 and L5 are available). (**a**) GPS; (**b**) GPS + GLONASS.

The performance of GNSS-only and GNSS/UWB system was assessed in open-sky environments based on one-hour static data. The cutoff elevation angle was set to 10°. The data processing was performed in four modes, namely GPS PPP mode, GPS + GLONASS PPP mode, GPS/UWB mode and GPS + GLONASS/UWB mode. The positioning difference between GNSS/UWB and GNSS-only solutions is analyzed. The number of available GNSS satellites and the corresponding positioning dilution of precision (PDOP) are shown in Figure 4. According to the statistics, the average satellite number for GPS and GPS + GLONASS are 6.7 and 12.9, and the corresponding PDOP values are 3.5 and 1.9, respectively. It is clear that the average number of visible GPS + GLONASS satellites is twice that of GPS satellites. Additionally, the PDOP of GPS + GLONASS remains at a lower level throughout the entire experimental process. The sky plots of GPS and GPS + GLONASS satellites during the experiment are depicted in Figure 5. Either GPS or the GLONASS system can provide good satellite continuity.

**Figure 6:**
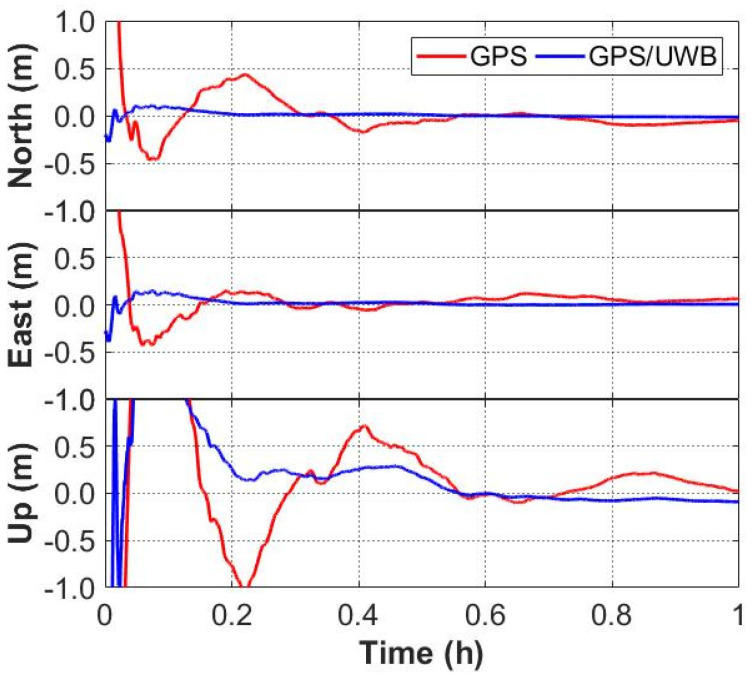
Positioning errors of GPS-only and GPS/UWB solutions.

**Figure 7:**
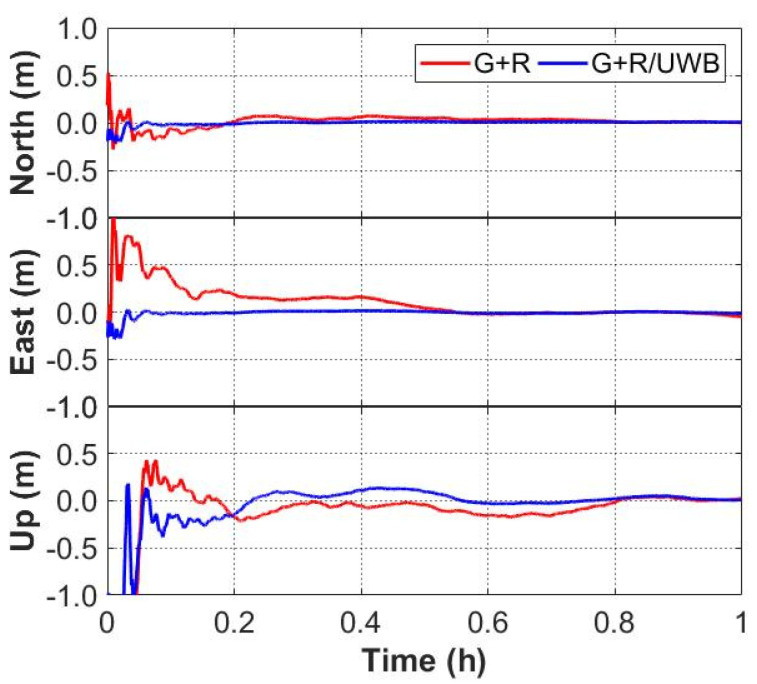
Positioning errors of GPS + GLONASS and GPS + GLONASS/UWB solutions.

The time series of the positioning errors for GNSS/UWB and GNSS-only solutions are shown in Figure 6 and Figure 7. It can be seen from Figure 6 that the positioning errors in the three directions with the GPS/UWB integration are obviously reduced when compared to the GPS-only solution. Similarly, when compared with the GPS + GLONASS solution, the time series of the positioning errors of GPS + GLONASS/UWB integration are smoother (Figure 7). As expected, compared with the GNSS-only solutions, the GNSS/UWB solutions have obvious improvements in positioning results and reach centimeter-level accuracy faster. The improvement in the north and east directions is more significant.

In this paper, the convergence criterion is defined as follows: the absolute value of the positioning errors is less than 0.1 m and remains within 0.1 m in the subsequent 300 epochs. The positioning errors of the three components after convergence are used for statistical RMS values. Table 1 summarizes the RMS values of the positioning errors with respect to GNSS/UWB and GNSS-only solutions. The results show that the RMS values of the GPS/UWB integration are 2.48 cm, 1.67 cm and 6.02 cm with an improvement of 4.46 cm, 5.98 cm and 5.20 cm in the north, east and up components, compared to that of the GPS-only solution. Besides, compared to the RMS values of positioning errors for the GPS + GLONASS solution, the RMS values of positioning errors for GPS + GLONASS/UWB integration show an improvement of about 2.72 cm, 1.68 cm and 2.23 cm in the north, east and up components. Regardless of whether GNSS-only solutions or GNSS/UWB solutions are used, all of them can achieve a centimeter-level positioning accuracy in horizontal directions after convergence. Additionally, the GNSS/UWB tightly coupled integration holds higher positioning accuracy. The RMS improvement percentages of the GNSS/UWB solutions with GNSS-only solutions in the north, east and up components are also shown in Table 1. In terms of positioning accuracy, the RMS values of the positioning errors for GPS/UWB integration in the north, east and up components are improved by 64.26%, 78.16%, and 46.34%, respectively. For multi-GNSS/UWB integration, the RMS values of the positioning errors are improved by 67.83%, 56.94%, 26.54% in the north, east and up components. Likewise, the improvement percentages are the most obvious in the horizontal direction.

Obviously, the combined utilization of GNSS and UWB data can improve the accuracy, stability and availability of PPP. This is the case because of a significant enhancement in the satellite availability and PDOP for multi-GNSS and sufficient measurement redundancy of UWB. Multi-constellation GNSS can increase the number of available satellites, which can lead to better continuity and geometric strength. The addition of the UWB system provides additional information and increases the number of observations. Besides, the enhancement in the vertical directions is small because the height of the UWB base stations was kept relatively stationary on a horizontal plane during the experiment.

#### 3.2.2. Static Results with a Simulation of Complex Environments

**Figure 8:**
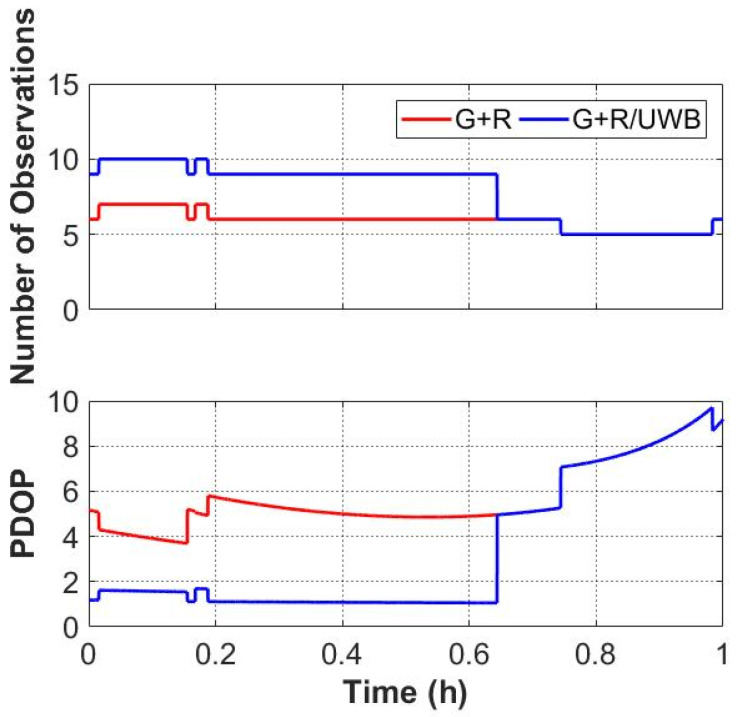
The number of observations (**top**) and the corresponding PDOP values (**bottom**) with a cutoff elevation angle of 40°.

The cutoff elevation angle was set to 40° to simulate an urban canyon. The static data were reprocessed, at points where the UWB signals were only present for about half an hour. The number of observations and the corresponding PDOP values are shown in Figure 8. Since only three GPS satellites are available most of the time, a GPS-only solution is not shown in the following. It can be seen that the number of observations increased when UWB signals were available. The GNSS-only solution has very poor PDOP values whereas the GNSS/UWB solution still has reasonable PDOP values.

**Figure 9:**
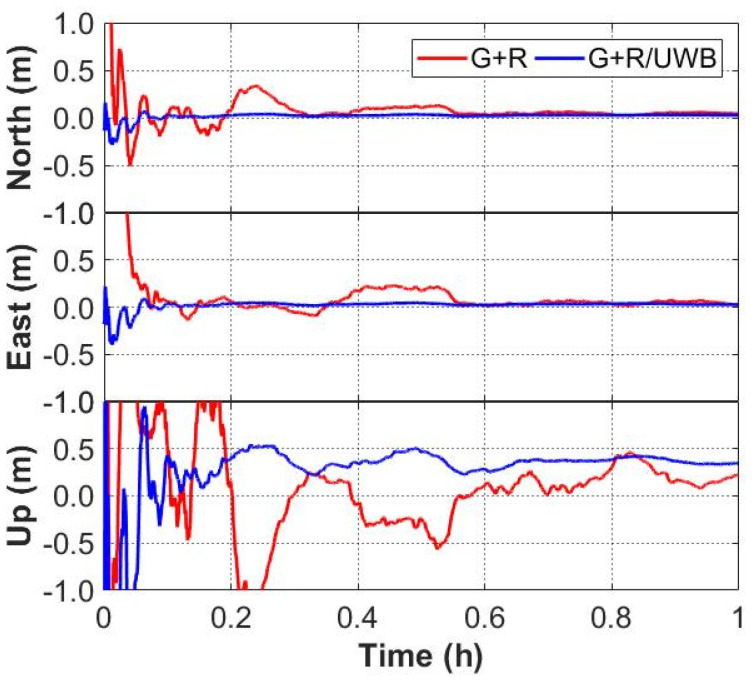
Positioning errors of GPS + GLONASS and GPS + GLONASS/UWB solutions with a cutoff elevation angle of 40°.

The time series of the positioning errors of GPS + GLONASS/UWB and GPS + GLONASS solution are shown in Figure 9. In a simulation of urban environments, the GPS + GLONASS/UWB solution has significant improvement in positioning results, with a smoother time series of the positioning errors compared with the GPS + GLONASS solution. The improvement in the north and east directions is still more dramatic, achieving centimeter accuracy within a few minutes. Table 2 provides a quantitative summary of the positioning errors RMS values for two solutions with a cutoff elevation angle of 40°. The GPS + GLONASS/UWB positioning results are 2.66 cm, 2.63 cm and 60.77 cm, which can achieve centimeter accuracy in the horizontal directions after convergence. The RMS values of the positioning errors of the GPS + GLONASS/UWB solution are improved by 58.50%, 74.26%, 6.86% in the north, east and up components when compared to the GPS + GLONASS solution. However, the error in the vertical direction is still large, which may be caused by the poor quality and insufficient number of satellites.

There are two main reasons for the above phenomenon. Firstly, when the satellite cutoff elevation angle is greater than 40°, the number of satellites and the geometric strength of the satellites decrease significantly (Figure 5). Secondly, when the UWB measurements are available, GNSS/UWB integration shows more redundancy and effectively improves the geometric structure of the observations.

### 3.3. Convergence Performance

**Figure 10:**
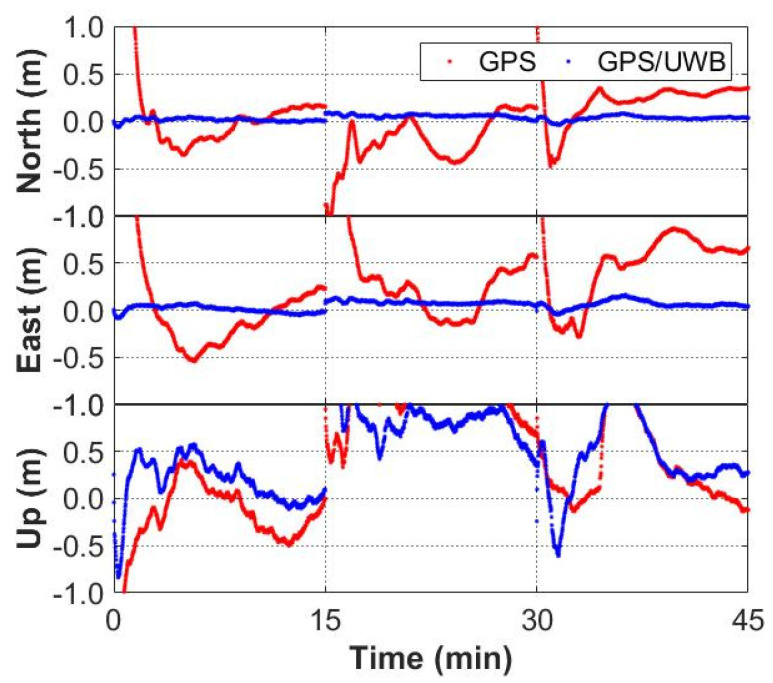
Convergence performance of GPS-only and GPS/UWB solutions for group A, B and C.

**Figure 11:**
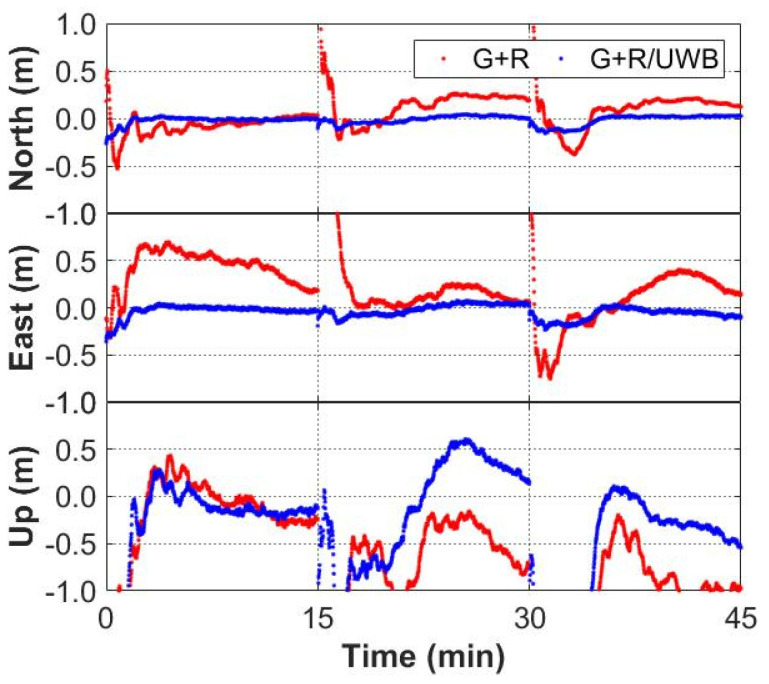
Convergence performance of GPS + GLONASS and GPS + GLONASS/UWB solutions for group A, B and C.

In order to evaluate the impact of the GNSS/UWB integration on PPP convergence performance, the static data was divided into three 15 min observation periods, namely group A, group B and group C. The GPS-only and multi-GNSS data of each group were processed with GNSS-only and GNSS/UWB solutions in kinematic mode. In the data processing, the EKF models the position error states as random walk processes with process noise suitable for the pedestrian environment. The results are shown in Figure 10 and Figure 11. The GNSS/UWB solutions can significantly improve the convergence time and perform convergence within a few minutes in the horizontal directions, while requiring more than 15 min in the vertical direction. The GNSS-only solutions seldom converge to centimeter-level accuracy within 15 min. Accordingly, the position accuracy could be improved by integrating GNSS and UWB in indoor-outdoor scenario, where GNSS signals are easy to block, especially in the horizontal direction.

### 3.4. Positioning Performance during GNSS Outage

To simulate the indoor scenario, the GNSS signals were interrupted when the trolley moved from Point 1 to Point 2, and the UWB signals were received at the same time. The set of dynamic data were processed to demonstrate the improvement of the positioning performance of the GNSS/UWB tightly coupled integration during GNSS signals outage. The interruption occurred at 7:15 and 7:22, lasting approximately one minute and 6 min, respectively.

**Figure 12:**
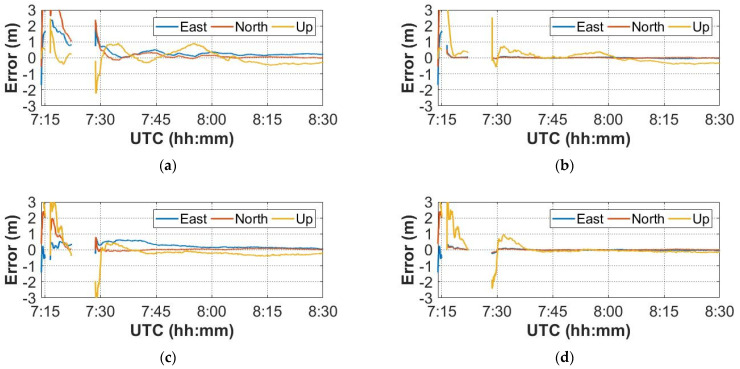
The positioning errors of GNSS-only and GNSS/UWB solutions during GNSS outage. (**a**) GPS PPP; (**b**) GPS/UWB; (**c**) GPS + GLONASS PPP; (**d**) GPS + GLONASS/UWB.

Figure 12 illustrates the positioning errors of the GNSS-only and GNSS/UWB solutions. After GNSS outage, the GNSS/UWB integration continues to work well, as can be seen in Figure 12b,d. The GNSS/UWB solutions achieve a decimeter-level accuracy in the initial stage, while GNSS-only solutions need to re-converge, and the initial convergence fluctuations in all components are large. In addition, the positioning errors of GPS-only solution are still large in the later epochs from Figure 12a. This may be caused by an insufficient convergence time. Figure 12c shows that the positioning errors of GPS + GLONASS solution is relatively small. Meanwhile, from Figure 12, we can also see that the GNSS/UWB tightly coupled integration can achieve high accuracy with a faster re-convergence by using both the multi-GNSS data and the UWB data.

The main reason is that the use of UWB sensors can increase the number of observations and provide additional external information for GNSS navigation. Therefore, it can also assist the system in maintaining better accuracy and significantly shortening the PPP re-convergence time.

## 4. Conclusions

This paper presents the tightly coupled integration positioning model of GPS, GLONASS and UWB systems. The positioning performance of the model has been validated by real-measured data from a trolley and a simulation of an urban environment. The results show that multi-GNSS/UWB integration can significantly improve the accuracy, continuity and stability of PPP. The positioning errors of the GNSS/UWB solutions in the horizontal directions can quickly reach a centimeter-level accuracy. In the open-sky environment, the RMS values of the positioning errors of GPS/UWB after convergence are 2.48 cm, 1.67 cm and 6.02 cm in the north, east and up directions, with 64.26%, 78.16% and 46.34% improvement when compared to GPS PPP. The RMS values of positioning errors of GPS + GLONASS/UWB are 1.29 cm, 1.27 cm and 6.17 cm in the three directions, with 67.83%, 56.94% and 26.54% enhancements when compared to GPS + GLONASS PPP. The convergence simulations show that the convergence time of the GNSS/UWB solutions is about a few minutes in the horizontal directions. The main reason is that the multi-GNSS can provide more available satellites and better spatial geometry structure, and the UWB system can also make up for the shortcomings of the GNSS-only solutions by increasing the number of observations and providing additional external information.

In addition, when the GNSS signals are interrupted, PPP faces the problem of re-convergence. This paper further analyzes the positioning performance of GNSS/UWB tightly coupled integration during GNSS outage. The GNSS outage simulations results indicate that the PPP convergence is faster with the addition of the UWB system. In summary, the positioning performance is significantly improved by tightly integrating GNSS and UWB, in terms of the stability, positioning accuracy and convergence time.

## Figures and Tables

**Table 1:** RMS values of the positioning errors of GNSS/UWB and GNSS-only solutions.

Items	GPS			GPS + GLONASS		
	North	East	Up	North	East	Up
GNSS-only (cm)	6.94	7.65	11.22	4.01	2.95	8.40
GNSS/UWB (cm)	2.48	1.67	6.02	1.29	1.27	6.17
Improvement (%)	64.26	78.16	46.34	67.83	56.94	26.54

**Table 2:** RMS values of the positioning errors for GNSS/UWB and GNSS-only solutions with a cutoff elevation angle of 40°.

Items	GPS + GLONASS		
	North	East	Up
GNSS-only solution (cm)	6.41	10.22	65.25
GNSS/UWB solution (cm)	2.66	2.63	60.77
Improvement (%)	58.50	74.26	6.86

## Data Availability

The data used in this study are available from the corresponding author.
